# Association of prehospital oxygen administration and mortality in severe trauma patients (PROMIS): A nationwide cohort study

**DOI:** 10.1097/MD.0000000000016307

**Published:** 2019-07-05

**Authors:** Yutaka Kondo, Koichiro Gibo, Toshikazu Abe, Tatsuma Fukuda, Ichiro Kukita

**Affiliations:** a Department of Emergency and Critical Care Medicine, Juntendo University Urayasu Hospital, Chiba; b Department of Emergency and Critical Care Medicine, Graduate School of Medicine, University of the Ryukyus; c Department of Emergency Medicine, Okinawa Chubu Hospital, Okinawa; d Department of General Medicine, Juntendo University, Tokyo; e Health Services Research and Development Center, University of Tsukuba, Tsukuba; f Department of Health Services Research, Faculty of Medicine, University of Tsukuba, Japan.

**Keywords:** oxygen administration, prehospital, propensity score, research design, trauma

## Abstract

Until now, we routinely administered oxygen to trauma patients in prehospital settings irrespective of whether oxygen delivery affected the prognosis. To determine the necessity of prehospital oxygen administration (POA) to trauma patients, we aimed to assess whether POA contributed to in-hospital mortality.

This was a multicenter propensity-matched cohort study involving 172 major emergency hospitals in Japan. During 2004 to 2010, 70,683 patients with trauma aged ≥15 years were eligible for enrolment. The main outcome measures were survival until hospital discharge after POA, and propensity score analyses were used to adjust for patient factors and hospital site.

Of 32,225 trauma patients, 19,985 (62.0%) were administered oxygen by the emergency medical services in prehospital settings and 12,240 (38.0%) did not receive oxygen. Overall, 29,555 patients (90.7%) survived till hospital discharge. In the multivariable unconditional logistic regression, POA had an odds ratio (OR) of 0.33 (95% confidence interval [CI], 0.30–0.37; *P* <.001) for favorable in-hospital mortality. Furthermore, there were significant differences in all the important variables between the POA and no POA groups (*P* <.001); therefore, we used propensity score matching analysis. After adjustment for the covariates of selected variables, we found that POA was not associated with a higher rate of survival after hospitalization (adjusted OR, 1.02; 95% CI, 0.99–1.04; *P* = .27). Even after adjustment for all covariates, POA did not improve in-hospital mortality (adjusted OR, 1.01; 95% CI, 0.99–1.03; *P* = .08).

In this study, POA did not improve in-hospital mortality in trauma patients. However, further studies are needed to validate our results.

## 1 Introduction

Trauma is a life-threatening and time-sensitive condition. Treatment in trauma patients is therefore commonly initiated even before hospital admission. Paramedics use various prehospital treatment methods such as prehospital oxygen administration (POA), cervical spine immobilization, and intravenous (IV) fluid administration. However, the effects of these procedures have not been validated. Recent research casts doubt on the efficacy of prehospital treatment.^[1,2]^ Studies have shown that prehospital endotracheal intubation does not improve mortality over bag-valve-mask ventilation in trauma patients, and instead leads to increased prehospital time.^[1,3]^ Another study reported that trauma patients who received prehospital IV fluids had higher mortality than those who did not receive IV fluids in the prehospital setting.^[2]^

In contrast, some prehospital interventions have been shown to reduce mortality.^[4,5]^ In particular, trauma patients receiving prehospital transfusion have shown improved mortality benefits.^[5,6]^ However, as many prehospital treatments were evaluated in these studies, the specific role of POA was not fully understood. The Advanced Trauma Life Support (ATLS) program, developed by the American College of Surgeons, is a popular training program for medical doctors for the management of acute trauma cases.^[7]^ As per the ATLS program, the airway is the first priority in trauma care. Hence, paramedics administer 100% oxygen through a mask to many patients in initial settings. Thus, it was believed that POA improves the outcome of trauma patients. However, there is a lack of evidence supporting the usefulness of this treatment. Hence, in this study, we aimed to clarify the relationship between POA and mortality in trauma patients.

## 2 Methods

### 2.1 Study design and data collection

This was a multicenter prospective observational study performed using data from the Japan Trauma Data Bank (JTDB). The JTDB was established in 2003 with the Japanese Association for the Surgery of Trauma (Trauma Registry Committee) and the Japanese Association for Acute Medicine (Committee for Clinical Care Evaluation) as the main parties. The aim of establishing the JTDB was to collect and analyze trauma data in Japan (patient and injury characteristics, information from emergency services, POA, vital signs at prehospital settings and at the first medical examination, inspections and treatments, diagnosis and injury severity score (ISS), information on discharge from the hospital, and mortality).

Data were collected at 172 major emergency hospitals in Japan that were chosen independently to participate in the registration. Approximately 40% of the participating institutions had resources equivalent to those in Level I trauma centers in the United States.^[8]^. Data were obtained from participating institutions via the internet.

### 2.2 Selection of participants

**Figure 1:**
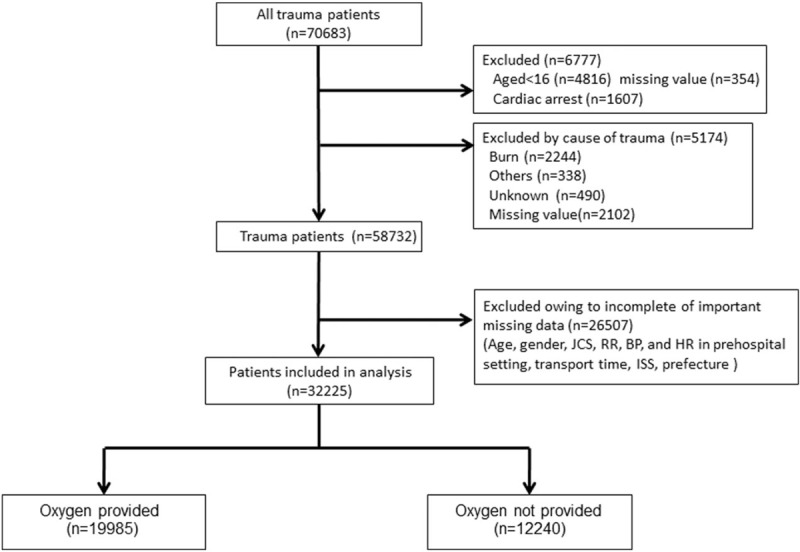
Flow chart of study population.

A total of 70,683 subjects registered in the JTDB from 2004 to 2010 were enrolled in this study. In total, 38,458 patients were excluded because they were 15 years of age or younger, had died during the initial examination at the scene, cause of trauma, or had missing data. Thus, 32,225 (45.6%) met the inclusion criteria with a complete data set on important variables for analysis (Fig. 1).

### 2.3 Variables and outcomes

For this study, we examined the following patient background characteristics: age, year, gender, prehospital systolic blood pressure, prehospital respiratory rate, prehospital pulse rate, prehospital Japan Coma Scale, situation major, transport time, Injury Severity Score, and Revised Trauma Score. The primary outcome in this study was in-hospital mortality, and the secondary outcome was prehospital mortality. We performed further sub-group analysis by dividing trauma patients into the head, chest, and abdominal trauma groups.

### 2.4 Statistical analysis

Continuous variables are presented as means and standard deviations (SDs), and categorical variables are presented as frequencies and percentages (Table 1). Patient variables were compared using Student *t* test for normal distribution of continuous variables, Mann–Whitney *U* test for the skewed distribution of continuous variables, and Fisher exact test for categorical variables.

**Table 1:**
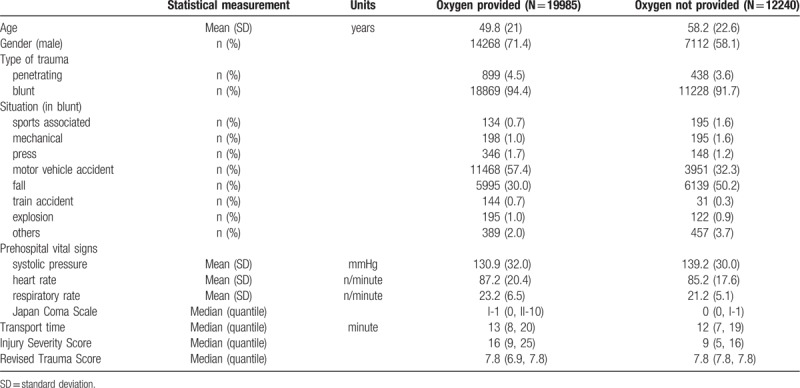
Baseline characteristics.

Propensity scores and propensity score matching, as well as all propensity score-based logistic regression analyses, were performed using “R 2.15.2” statistical analysis software (http://www.r-project.org/).^[9]^ With 90% power and α level of 0.05, a total of 8802 patients were required to detect a 2% mortality difference between groups. The 2-sided significance level for all tests was *P* <.05.

The protocol for the present study was approved by the Ethics Committee of the University of the Ryukyus.

## 3 Results

The main characteristics of the trauma patients are shown in Table 1. Between 2004 and 2010, 70,683 trauma patients were registered and of these, 32,225 met the inclusion criteria (Table 1 and Fig. 1). The mean age of all patients with POA and without POA was 49.8 (SD, 21) years and 58.2 (SD, 22.6) years, respectively, and a significant difference existed between the mean ages of the 2 groups (*P* <.01). Therefore, we adjusted the significant difference by using propensity-matched score analysis presented in Table 2 (adjusted covariates: age, year, gender, prehospital systolic blood pressure, prehospital respiratory rate, prehospital pulse rate, prehospital Japan Coma Scale, situation major, and transport time).

**Table 2:**
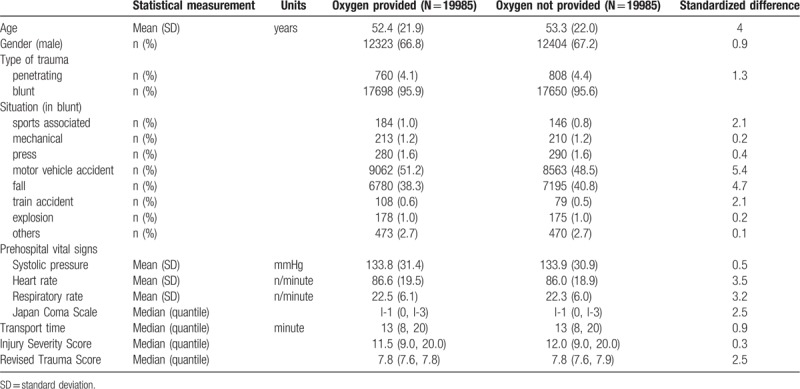
Baseline characteristics in propensity matched patients.

**Figure 2:**
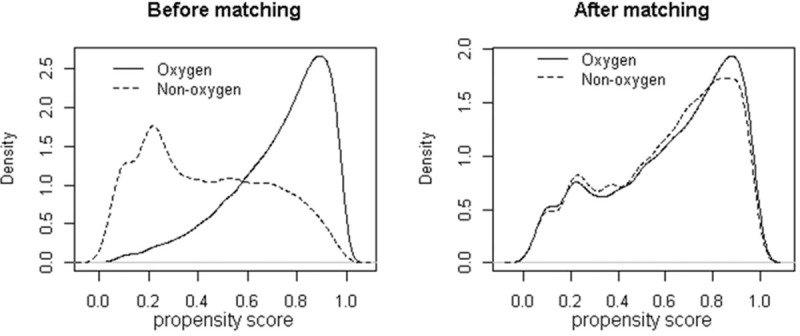
Density plot of propensity scores of trauma patients with (dotted line)/without (solid line) POA. The overlapping area represents trauma patients with similar propensity scores available for close matches. POA = prehospital oxygen administration.

Figure 2 displays the density plot of the propensity score of trauma patients who met the inclusion criteria. Before matching, there were a few overlapping areas between POA and no POA. However, after matching, the shapes were almost similar. It means that propensity score matching leads to a proper adjustment in characteristics of trauma patients with or without POA.

**Figure 3:**
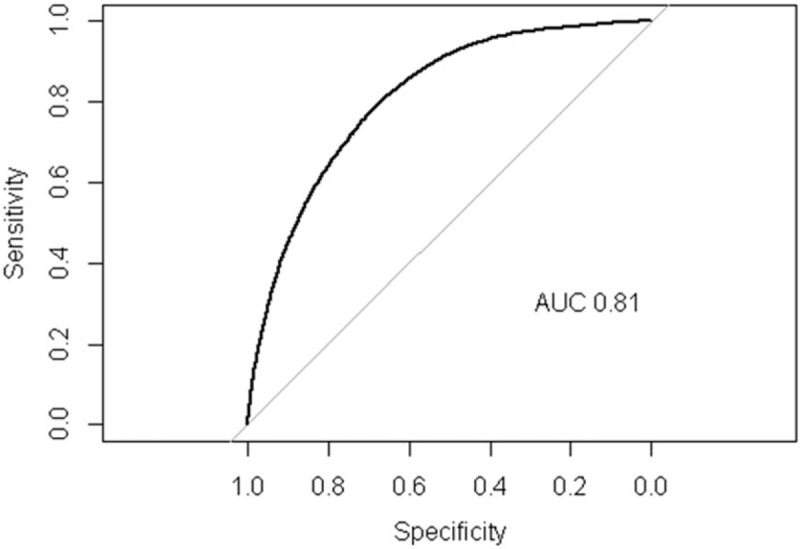
The AUC for fitting of the propensity scores. AUC = area under the curve.

Figure 3 shows the receiver operating characteristic curve in this model. The value of the area under the curve was 0.81 which meant a good fit under these conditions.

**Figure 4:**
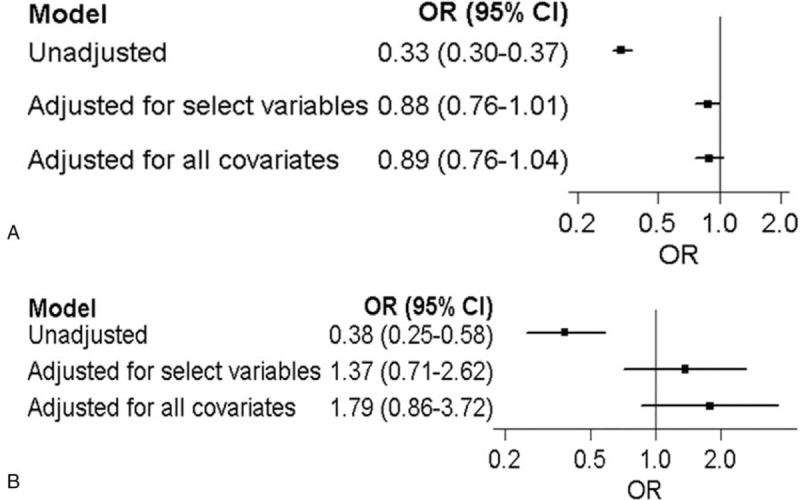
(A) In-hospital mortality of included trauma patients. (B) Prehospital mortality of included trauma patients.

Figure 4A summarizes in-hospital mortality until discharge from the hospital (long-term mortality). A significant negative association was observed in the crude model (odds ratio [OR], 0.33; 95% confidence interval [CI], 0.30–0.37; *P* <.01); however, no significant associations in the adjusted model using selected variables (OR, 0.88; 95% CI, 0.76–1.01; *P* <.01) or all variables (OR, 0.89; 95% CI, 0.76–1.04; *P* <.01) were observed for survival at discharge between POA and in-hospital mortality.

Figure 4B shows prehospital mortality until arrival at the hospital (short-term mortality). A significant negative association was observed in the crude model (OR, 0.38; 95% CI, 0.25–0.58; *P* <.01); however, no significant associations in the adjusted model using selected variables (OR, 1.37; 95% CI, 0.71–2.62; *P* <.01) or all variables (OR, 1.79; 95% CI, 0.86–3.72; *P* <.01) were observed for survival at discharge between POA and in-hospital mortality.

**Figure 5:**
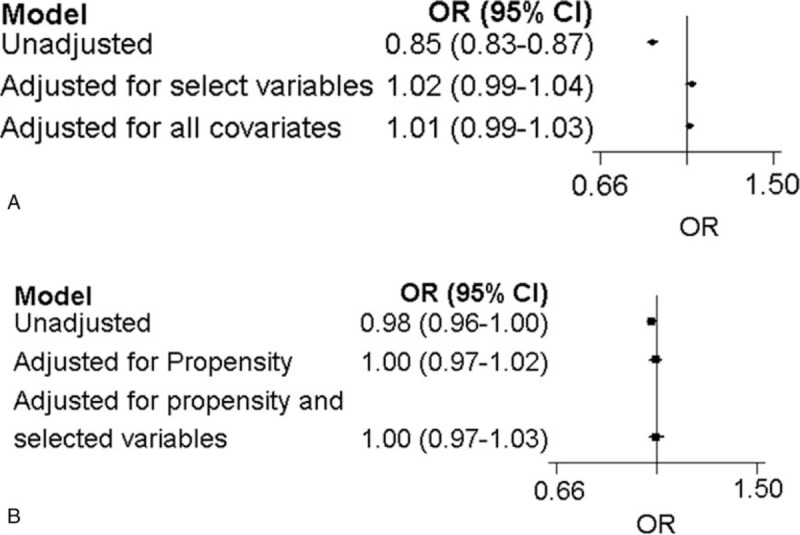
(A) In-hospital mortality after propensity score matching. (B) Prehospital mortality after propensity score matching.

Demographic characteristics were similar between the propensity-matched groups. Figure 5A and B summarize the survival outcomes by POA among propensity-matched patients. The unadjusted model showed significant negative associations between POA and the mortality measures (*P* <.01 for all). In the multivariable models using selected and all variables, significant negative associations were detected between POA and the endpoint. Figure 5A shows significant negative association in the crude model (OR, 0.85; 95% CI, 0.83–0.87; *P* <.01); however, no significant associations in the adjusted model using selected variables (OR, 1.02; 95% CI, 0.99–1.04; *P* <.01) or all variables (OR, 1.01; 95% CI, 0.99–1.03; *P* <.01) were observed for survival at discharge between POA and in-hospital mortality. Figure 5B showed a significant negative association in the crude model (OR, 0.98; 95% CI, 0.96–1.00; *P* <.01); however, no significant associations in the adjusted model using selected variables (OR, 1.00; 95% CI, 0.97–1.02; *P* <.01) or all variables (OR, 1.00; 95% CI, 0.97–1.03; *P* <.01) were observed for survival at discharge between POA and in-hospital mortality.

As observed in Table 3, POA showed no significant effects in the head, chest, and abdominal sub-groups after propensity-matched adjustments.

**Table 3:**
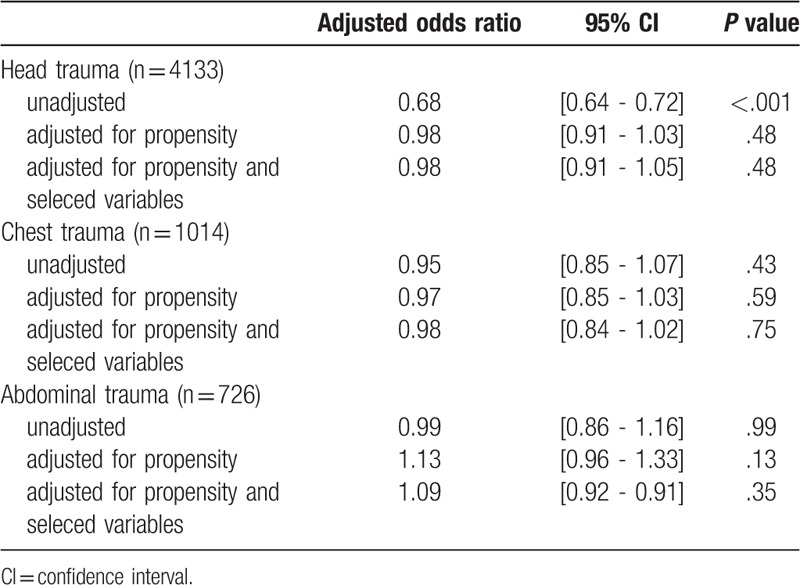
Subgroup analysis based on injury location.

## 4 Discussion

The aim of our study was to clarify the significance of POA, which is routinely performed in initial clinical settings. In our study, POA did not improve both on-arrival and in-hospital mortality in trauma patients.

We presumed that POA could increase SpO_2_ levels temporarily; however, it was not associated with improvement in mortality in trauma patients. In fact, almost all trauma patients died of hemorrhagic shock and not hypoxia. For this reason, many attempts at improving the mortality rate in trauma patients include treatment of abnormality in “Circulation” of the ‘Airway,” “Breathing,” “Circulation,” “Disability,” and “Environment and exposure” (ABCDE) approach.^[10–12]^ In addition, trauma patients with excessive POA could have a worse outcome because of the harmful effect of oxygen therapy.^[13,14]^ Oxidative stress, which is characterized by an imbalance between reactive oxygen species and the antioxidative defense system, results in the production of free radicals at a rate that is far higher than the rate at which the body is able to eliminate them. This mechanism may lead to worsening the situation by POA in some trauma patients.

As mentioned earlier, many prehospital treatments, such as prehospital endotracheal intubation and prehospital intravenous fluid administration in trauma patients were regarded as inconsequential. This suggests that fast, definitive treatment is most important in trauma patients. The POA group had a significantly longer transportation time (by 1 min) than the no POA group. Paramedics must prepare oxygen masks and bottles and adjust the flow by considering patients’ SpO2 levels, which makes them stay on the scene for a prolonged duration.

However, it does not mean that POA is completely meaningless; we have to consider other factors such as wound healing, length of morbidity, and neurological outcome. Especially, traumatic brain injury without injury to other parts of the body allows patients to have a chance to improve their neurological outcome.

The strength of the study is that propensity-matched score analysis has been used. A previous study has verified the usefulness of this propensity-matched score analysis in prehospital trauma care research.^[7]^

There are several limitations to be considered when interpreting the results of our study. We used only 45.6% of eligible patients for analysis largely because of missing data. Thus, there might be a selection bias.

Next, we used a national standard covariate; the Japan Coma Scale (JCS) instead of the Glasgow Coma Scale. Since Japanese paramedics in emergency medical services can use only the JCS scale, the JTDB has no GCS data in prehospital settings. JCS is a 10-grade scale of consciousness and it is not an optimized scale as compared to the GCS. There may be some information bias if we analyze other types of datasets. However, in a previous JTDB study, the accuracy of the JTDB data ware validated by using Trauma Audit and Research Network and other trauma registry data.^[15–17]^ Therefore, we concluded that it did not influence the main results.

Finally, our results were based on a propensity score-matched analysis. Before using propensity score analysis, there were remarkable differences in backgrounds among patients with or without POA. Several factors are associated with the decision to provide POA. However, the propensity score adjusted the backgrounds well by which we could select important covariates associated with mortality. In addition, to overcome the effects of not selecting important variables, we performed “Rosenbaum sensitivity analysis” (data not shown). It can detect hidden bias arising from unobserved variables and the study approved the results. If we could conduct a prospective study, the results will be more clearly illustrated. However, considering ethical issues, we may not be able to examine prospectively whether POA improves mortality in trauma patients.

## 5 Conclusions

Prehospital oxygenation could not improve mortality in severe trauma patients by using propensity score analysis. The neurological outcomes were not known, and further validation of our results is required.

## Acknowledgments

The authors would like to thank all the paramedics, emergency medical technicians, nurses, and physicians who participated in the JTDB study.

## Author contributions

**Conceptualization:** Yutaka Kondo.

**Data curation:** Yutaka Kondo.

**Formal analysis:** Koichiro Gibo.

**Methodology:** Yutaka Kondo, Toshikazu Abe, Tatsuma Fukuda.

**Project administration:** Ichiro Kukita, Yutaka Kondo.

**Supervision:** Ichiro Kukita.

**Validation:** Toshikazu Abe.

**Writing – original draft:** Yutaka Kondo.

**Writing – review & editing:** Tatsuma Fukuda.
